# Giant multilocular prostatic cystadenoma

**DOI:** 10.1186/s12957-019-1579-7

**Published:** 2019-02-26

**Authors:** Yuya Nakamura, Dai Shida, Takahiro Shibayama, Akihiko Yoshida, Yoshiyuki Matsui, Yasuo Shinoda, Shintaro Iwata, Yukihide Kanemitsu

**Affiliations:** 1 Department of Colorectal Surgery, National Cancer Center Hospital, 5-1-1 Tsukiji, Chuo-ku, Tokyo 1040045 Japan; 2 Urology Division, National Cancer Center Hospital, Tokyo, 1040045 Japan; 3 Department of Musculoskeletal Oncology and Rehabilitation, National Cancer Center Hospital, Tokyo, 1040045 Japan; 4 Pathology and Clinical Laboratory Division, National Cancer Center Hospital, Tokyo, 1040045 Japan

**Keywords:** Giant multilocular prostatic cystadenoma, Retroperitoneal tumor, Prostatic tumor

## Abstract

**Background:**

The giant multilocular prostatic cystadenoma is a very rare benign tumor of the prostate gland. It is composed of predominantly cystic enlarged prostatic glands in a fibrous stroma and spreads extensively into the pelvis. Because of the large size at the time of diagnosis, it is not always possible to determine the exact point of origin for these multilocular cystic neoplasms. Thus, diagnosis before histological examination of a surgical specimen is often difficult. Here, we present a case involving one of the largest giant multilocular prostatic cystadenomas reported in the literature and discuss preoperative diagnoses and appropriate surgical approaches for this rare retroperitoneal tumor.

**Case presentation:**

A 50-year-old man presented with a 2-year history of abdominal distension and lower urinary symptoms. Enhanced CT showed a large retroperitoneal mass with multiple septations in the pelvis and lower abdomen, measuring 30 cm in size, surrounding the rectum and displacing the bladder, prostate, and seminal vesicle to the right anterior side. MRI showed multiple cysts with a simple fluid appearance on T2-weighted images and enhanced solid components on gadolinium-enhanced fat-saturated T1-weighted images, suggesting the retroperitoneal mass as leiomyoma with cystic degeneration or perivascular epithelioid cell tumor. Biopsy of the mass showed a spindle cell tumor with focal smooth muscle differentiation. Differential diagnosis comprising leiomyoma, low-grade leiomyosarcoma, and perivascular epithelioid cell tumor was made. Complete resection of the tumor with low anterior resection of the rectum was performed. The tumor was solid with multilocular cavities containing blackish-brown fluid and measured 33 × 23 × 10 cm. Histologically, the tumor was composed of variously sized dilated glandular structures lined by prostatic epithelia surrounded by fibromuscular stroma. The prostatic nature of the lesions was confirmed by immunohistochemical staining of the epithelium for prostate-specific antigen. Thus, pathological diagnosis was a giant multilocular prostatic cystadenoma.

**Conclusions:**

We present our experiences with one of the largest giant multilocular prostatic cystadenomas. When a retroperitoneal huge lesion with locular cavities fills the pelvis in a male patient, the possibility of giant multilocular prostatic cystadenoma should be considered before planning for retroperitoneal tumor treatment.

## Background

Retroperitoneal tumors often present diagnostic and therapeutic challenges. Although computed tomography (CT) and magnetic resonance imaging (MRI) may be useful for diagnosis and understanding the extent of disease, a definitive diagnosis can be established only by histopathologic analysis after surgery. Knowledge of the tumor characteristics may help to narrow down the differential diagnosis. Here, we report a case of a giant multilocular prostatic cystadenoma, an extremely rare benign neoplasm that originates in the prostate glands. Fewer than 30 cases have been reported thus far [1–21]. Our case was one of the largest reported to date, as it measured 33 cm in length and occupied the whole pelvis up to the level of the navel.

## Case presentation

A 50-year-old man presented with a 2-year history of abdominal distension. He also had lower urinary symptoms such as the sensation of incomplete voiding and increased frequency. He had no symptoms of bowel obstruction. Physical examination revealed a palpable mass occupying the lower abdomen up to the level of the navel, but there was no tenderness. Digital rectal examination revealed an elastic hard mass on the anterior side of the rectum without palpable intraluminal mass. Total colonoscopy showed no masses or stenosis in the rectum. We evaluated the urinary symptoms were due to the compression of the bladder by the tumor.

**Fig. 1:**
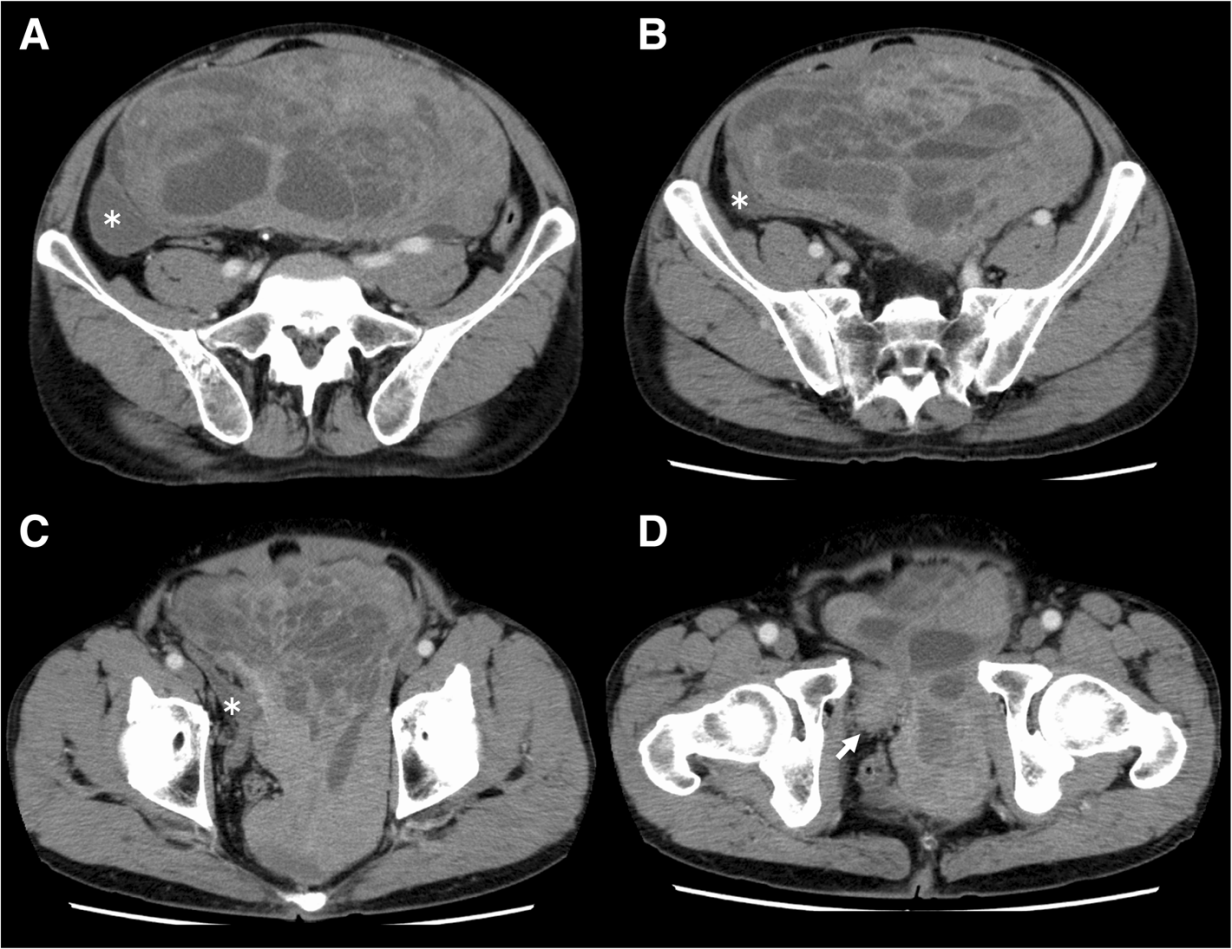
Enhanced abdominal CT. **a**, **b**, **c** A large multilocular mass with soft tissue component displacing the bladder (*) and rectum anterolaterally. **d** The arrow shows the prostate

**Fig. 2:**
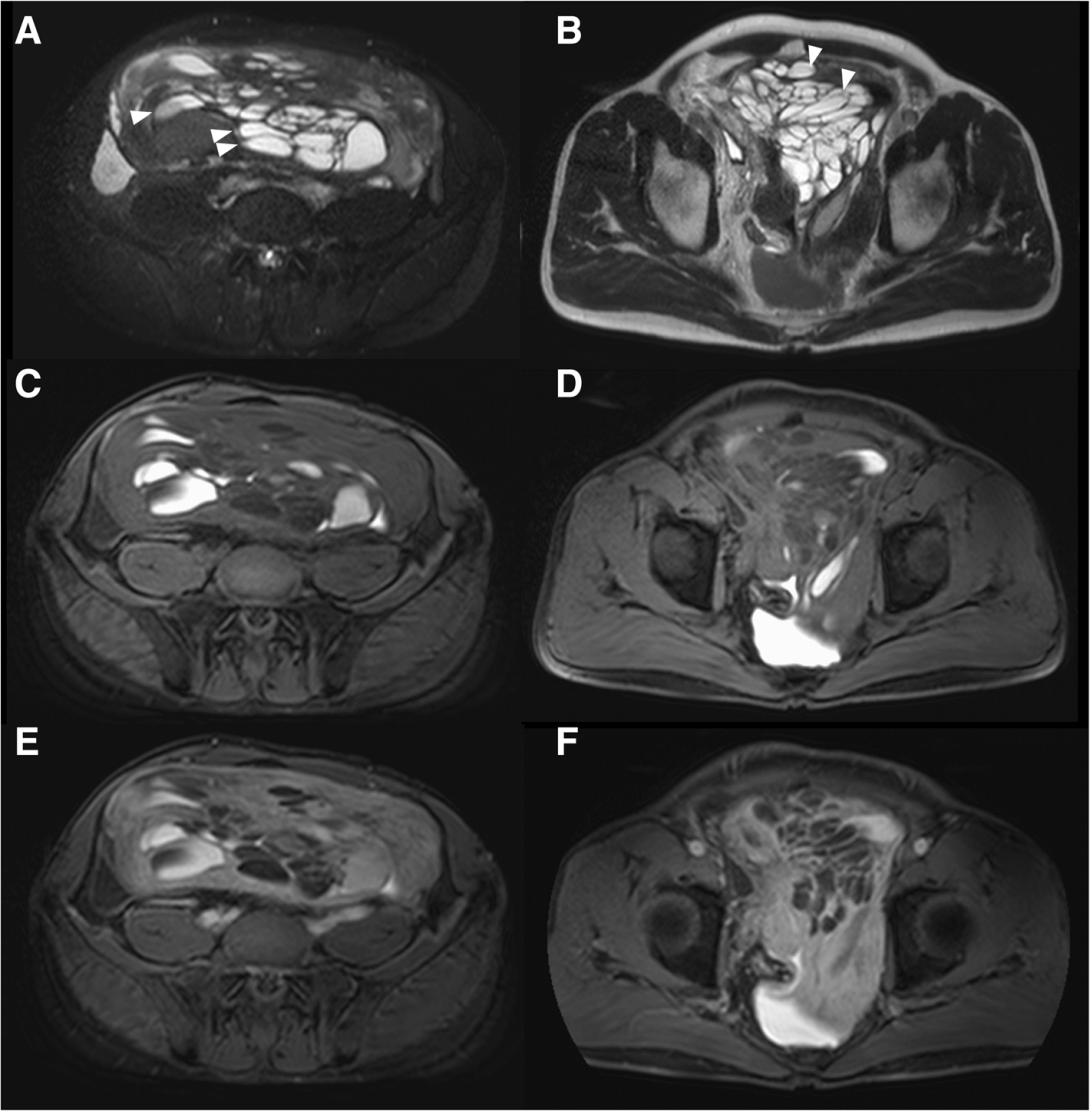
**a**, **b** T2-weighted MRI. Most cysts showed high intensity suggesting simple fluid, and some showed the presence of layering which suggests the likelihood of either fat or blood in content. The arrowheads show the presence of layering within the cystic areas. **c**, **d** Non-enhanced fat-saturated T1-weighted MRI and **e**, **f** gadolinium-enhanced fat-saturated T1-weighted MRI. Several solid components were enhanced

The results of laboratory tests were normal. Serum prostate-specific antigen (PSA) was not available preoperatively. Urinalysis was normal, with no evidence of hematuria. Enhanced CT showed a large retroperitoneal mass measuring 30 cm in size with multiple septations surrounding the rectum and displacing the bladder, prostate, and seminal vesicle to the right anterior side (Fig. 1). MRI showed a mass composed of cysts of various sizes ranging from smaller than 1 up to 6 cm and solid components. Whereas most cysts had simple fluid appearance (very high intensity on T2-weighted images), some showed the presence of layering which suggests the likelihood of either fat or blood in content (Fig. 2a, b). Several solid components which showed isointensity on T2-weighted images were enhanced on gadolinium-enhanced fat-saturated T1-weighted images (Fig. 2c–f). From these radiological findings, preoperative diagnosis was leiomyoma with cystic degeneration or perivascular epithelioid cell tumor (Fig. 2). Biopsy of the mass was performed under CT guidance, and histology showed a spindle cell tumor. Immunohistochemically, preoperative biopsy of the tumor showed positive staining for SMA, desmin, and caldesmon while negative for S-100, HMB-45, and MDM2, indicating smooth muscle differentiation. Differential diagnosis of leiomyoma, low-grade leiomyosarcoma, and perivascular epithelioid cell tumor was made. We suspected the tumor originated from the smooth muscle of the bladder or deep soft tissue in the retroperitoneal space.

As the biopsy findings did not reveal obvious evidence of malignancy and because the outer wall of the tumor was relatively smooth, we chose to forego total pelvic exenteration and instead attempt complete macroscopic resection of the tumor with minimal combined resection of adjacent organs.

**Fig. 3:**
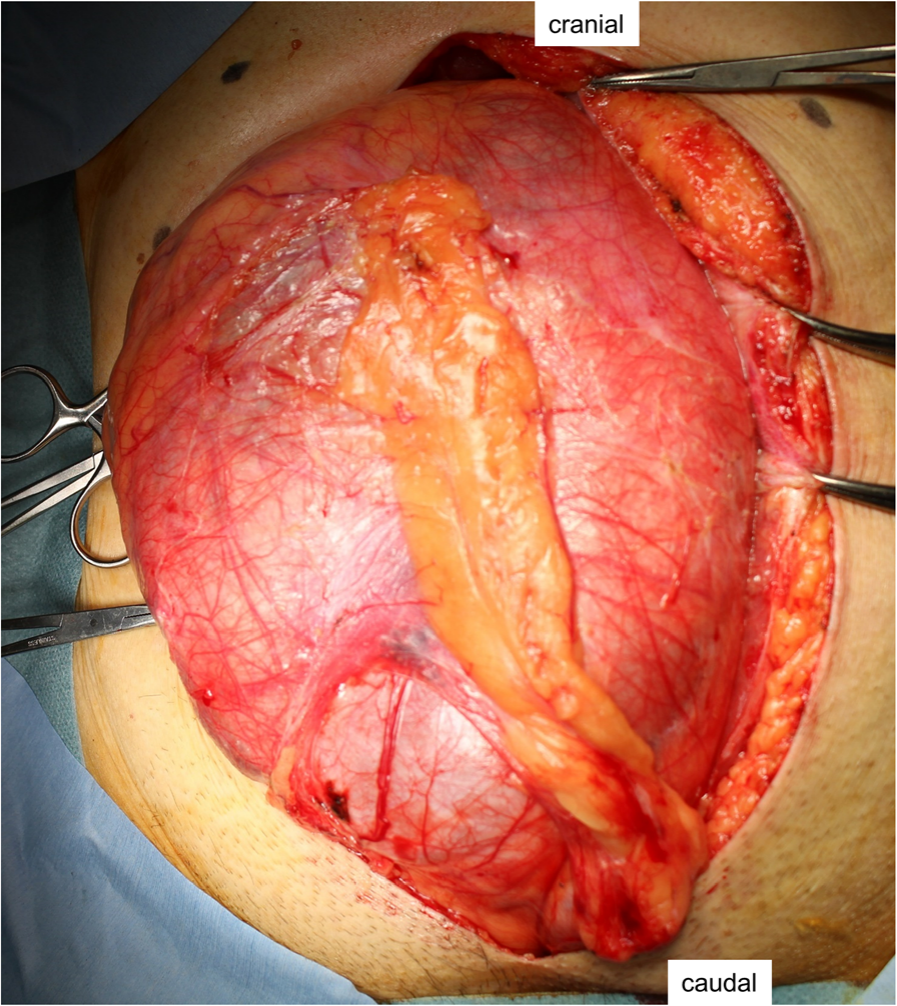
Intraoperative view. Large mass with a smooth surface in the lower abdomen and pelvis

Laparotomy was performed through a midline incision, which revealed a huge mass from the pelvic floor up to the level of the navel (Fig. 3). During exploration, the bilateral ureter was preserved but the vas deferens was resected. Because the plane of the interface between the bladder and tumor was unrecognizable, we injected air into the bladder and identified the border of the mass. We dissected the tumor, preserving the bladder and prostate with partial resection of the prostate. The tumor was not attached to the sacrum or levator ani and was mobilized from the pelvic floor. Because the tumor extended into the left pelvic sidewall and surrounded the rectum, we sacrificed the rectum as well as the left hypogastric nerve and left pelvic plexus. Finally, we performed a complete resection of the tumor with low anterior resection. Intraoperatively, the border between the tumor and normal prostate was not so clear.

**Fig. 4:**
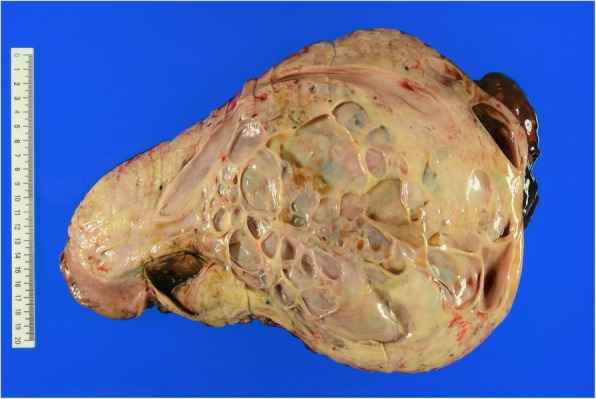
Gross photograph. Sectioning revealed a multicystic cut surface

**Fig. 5:**
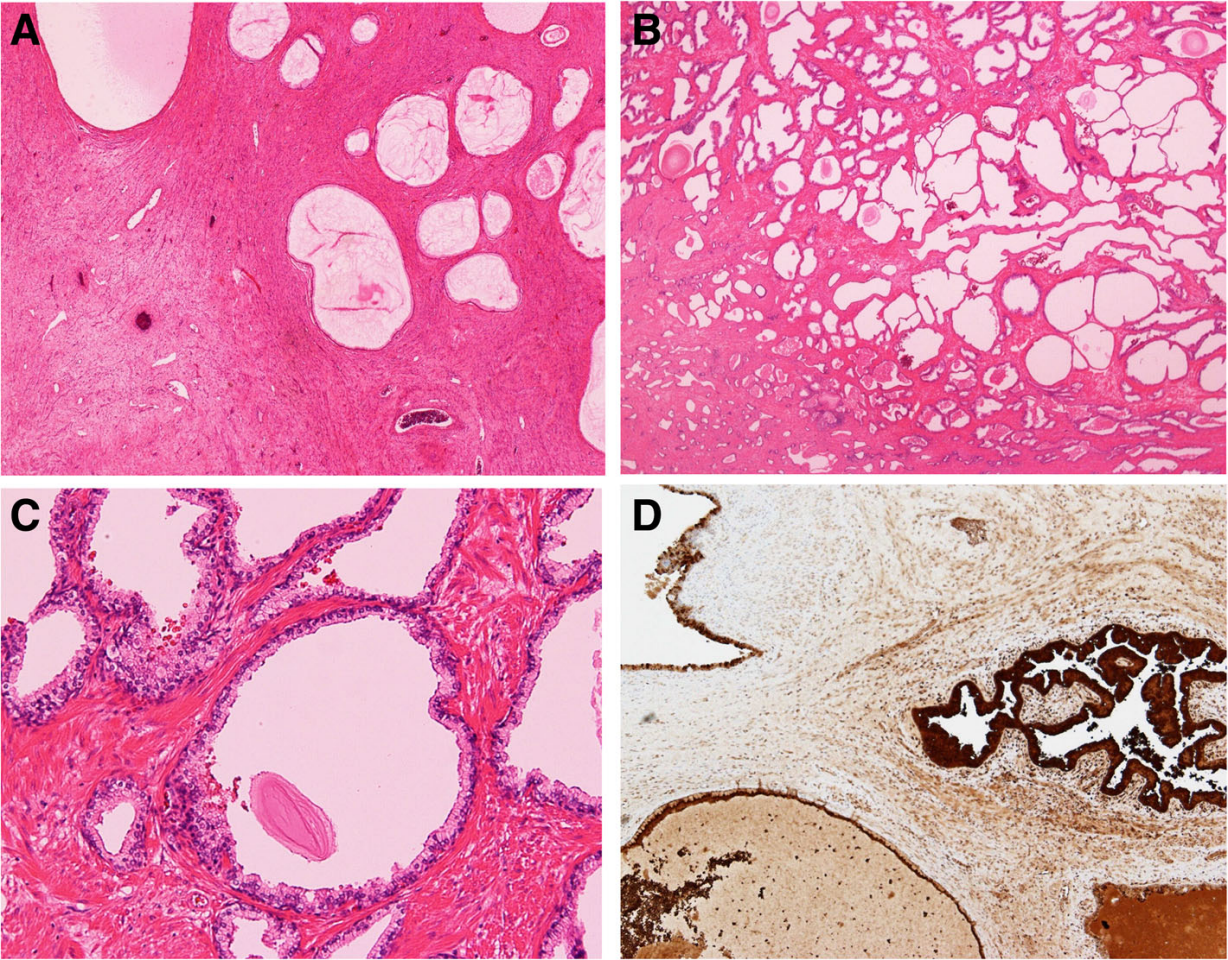
Pathological examination of the resected specimen. **a** Low-power magnification of cystadenoma of the prostate with dense fibromuscular stroma (H&E, original magnification × 20). **b** Cystic dilated glandular structures surrounded by a fibrous stroma resembling prostatic hyperplasia (H&E, original magnification × 20). **c** Glands and cysts are lined by cuboidal to low columnar epithelial cells with basally located nuclei (H&E, original magnification × 40). **d** The epithelium of the cysts stained positively with prostate-specific antigen stain (× 20)

The gross pathologic specimen was a 33 × 23 × 10 cm solid mass containing multilocular cavities. Sectioning revealed a multicystic cut surface (Fig. 4), and blackish-brown intratumoral fluid was drained from the tumor. Histologically, the tumor was composed of variously sized dilated glandular structures lined by prostatic epithelia surrounded by fibromuscular stroma (Fig. 5a). Lesions of the well-developed, dilated prostate glands resembling prostatic hyperplasia were also evident (Fig. 5b). The cysts were lined by cuboidal to columnar secretory cells and basally located nuclei (Fig. 5c). Cytologically, we observed no atypical features or mitosis. Epithelial cells of the cysts and stromal glands were positive for PSA on immunohistochemical staining (Fig. 5d). The epithelium cells of the tumor were also positive for AR and NKX3.1 staining, indicating that the tumor originated from the prostate. The spindle cells seen on preoperative biopsy were thought to be stromal components of the tumor.

Final histology indicated a giant multilocular prostatic cystadenoma.

Postoperatively, whereas the patient developed a pelvic abscess due to urine leakage from the prostatic urethra, he recovered conservatively and was discharged on postoperative day 37. Since there is no positive evidence of adjuvant therapy of this prostatic cystadenoma, the patient is now under follow-up by blood tests and CT scan every 3–6 months.

At 2 months after surgery, the patient had a PSA of 0.365 ng/ml, which was within the normal ranges. Since then, we have observed no signs of tumor recurrence. The patient was completely asymptomatic at the 6-month follow-up visit and made a full recovery, with no complaints of any urinary disorders or sexual dysfunction.

## Discussion and conclusions

A giant multilocular prostatic cystadenoma is an extremely rare benign neoplasm that originates in the prostate glands. In 1991, Maluf et al. became the first to describe and designate this rare entity [2]; since then, fewer than 30 cases have been reported (Table 1) [1–21]. This type of tumor is typically located along the midline between the bladder and rectum and identified as a large retroperitoneal mass. A definitive diagnosis before histological examination of a surgical biopsy is very difficult, but preoperative assessment is important for surgical treatment planning. Previous reports of giant multilocular prostatic cystadenomas indicate that surgical treatment varies from tumor debulking to total pelvic exenteration. The decision concerning surgical margin is influenced by whether or not the mass is malignant in nature and whether it has invaded adjacent organs. Information on the origin of the mass is also necessary in order to preserve adjacent organs.

**Table 1:** Cases of giant multilocular prostatic cystadenoma

Reference	Year	No. of cases	Age (years)	Clinical symptoms	PSA (ng/ml)	Biopsy	Radiologic findings	Size (cm)	Volume (g)	Contents	Treatment	Folllow-up	Outcome
Watanabe et al.	1990	1	80	urinary retention	8.2	connective tissue	large tumor in the pelvic cavity	11 × 8 × 7	660	cloudy fluid similar to semen	anterior pelvic exenteration	2 years	no recurrence
Maluf et al.	1991	2	28	Urinary retention	NA	Fragments of prostatic tissue	NA	19 × 16 × 7	600	Brown fluid and others with gray color	Surgical excision	4 months	No recurrence
			38	Increasing abdominal girth, difficulty in micturition	NA	NA	NA	45 × 35 × 13	6500	Serous, serosanguinous, or mucinous fluid	Resection → exenteration and pelvic irradiation	2 years	No recurrence
Lim et al.	1993	1	64	Lower abdominal pain, obstructive voiding symptoms	NA	NA	Fluid-filled mass	17 × 13 × 7	NA	NA	En bloc excision	NA	NA
Levy et al.	1993	1	56	Decreased force of urinary stream, hesitancy and frequency of urination	NA	Negative for malignant cells (cytology)	Multi-septated pelvic mass with thickened irregular walls	13 × 10 × 7	NA	Yellowish-brown fluid	Complete surgical excision	8 years	No recurrence
Morimoto et al.	1994	1	45	Urinary retention	6	Benign prostatic tissue	A mass with multilocular structure at the position of the prostate	5.5 × 5 × 5	210	Yellow fluid	Surgical resection	9 months	No recurrence
Kirsch et al.	1996	1	65	Hemorrhoidal pain, obstructive voiding symptoms, gross hematuria	30.2	No evidence of malignancy (cystic component)	Heterogeneous mass arising from the prostate and extending to the level of the sacral promontory	15 × 12.5 × 3	130	Straw-colored fluid	Full enucleation	1 year	No recurrence
Choi et al.	2000	1	57	Lower abdominal mass and dysuria	NA	NA	Large multiseptated pelvic mass with thickened irregular walls	15 × 10 × 8	300	Brown, rusty appearing fluid, transparent serous fluid	Surgical resection	NA	NA
Seong et al.	2002	1	48	Gross hematuria and frequency	68.2	Chronic inflammatory lesion	Oval, low-attenuated mass between the bladder and rectum, containing cystic and solid portions	8 × 7.5 × 6	180	Reddish-brown serous fluid	Excision with bilateral seminal vesicles and vas deferens	NA	No recurrence
Matsumoto et al.	2002	1	35	Gross hematuria	14.4	NA (abandoned)	Multilocular mass replacing the prostatic gland	9 × 8 × 6	860	Reddish-brown serous fluid	Radical cystoprostatectomy	24 months	No recurrence
Rusch et al.	2002	2	30	Urinary retention, lower urinary tract symptoms	NA	Benign prostatic tissue with cystic dilatation of glands and no evidence of malignancy	Multiple septations with some soft-tissue components	15	NA	Hemorrhagic fluid	Surgical resection	18 months	No recurrence
			41	Urinary retention (recurrence)	NA	NA	Multiseptated cystic mass	15	NA	NA	Surgical excision	NA	NA
Datta et al.	2003	1	71	Urinary retention	NA	NA	Cystic pelvic mass	12 × 7 × 4.5	NA	NA	Cystoprostatectomy and removal of a portion of the rectum, GnRH antagonist	11 years	Recurrence and hormonal therapy
Allen et al.	2003	1	52	Lower urinary tract symptoms, gross hematuria	3	NA	Multiloculated cystic pelvic mass	14 × 10 × 1	NA	NA	Completely removed piecemeal with the largest tissue fragment	NA	NA
Ganesan et al.	2006	1	28	Reduced force of the urinary stream and hesitancy and frequency of urination	1.6	Cystic lesion lined by columnar epithelium with cystic dilatation of prostatic acini	Cystic mass with multiple thin, echogenic internal septations (transabdominal ultrasound)	9.9 × 8.5 × 7.3	NA	Straw-colored fluid	Debulking	NA	NA
Tuziak et al.	2007	2	42	Obstructive urinary symptoms, urinary retention	0.04	Fibrous tissue interspersed with prostatic glands having no atypical features	Multicystic pelvic mass	15	NA	NA	Open prostatectomy without seminal vesicle removal	2 years	No recurrence
Park et al.	2007	1	61	Abdominal distension, urinary retention, aspermia	38.2	NA	Mass located between the rectum and bladder	9 × 7	NA	NA	Mass excision	1 year	No recurrence
Chowdhury et al.	2009	1	35	Left loin pain, hematuria, micturition	NA	NA	Complex septated cystic/soft tissue mass	20 × 11 × 15	NA	NA	Debulking	NA	NA
Lee et al.	2010	1	71	Urinary tract obstruction	NA	NA	Multilocular heterogenous cystic pelvic mass posterior to the bladder and anterior to the rectum	10 × 6.5 × 5.5	NA	Dark brown hemorrhagic fluid	Radical cystoprostatectomy	1 year	No recurrence
Olgun et al.	2012	1	23	Obstructive voiding symptom, difficulty in defecation	20.2	Only red blood cells and histiocytes with groups of benign epithelial cells	Multiseptated, huge cystic lesion that filled the pelvis completely	7 × 5 × 2.5 solid mass, 9 × 9 × 0.2 cystic component	NA	Serous and mucinous fluids	Debulking of the mass	18 months	No recurrence
Baad et al.	2015	1	55	Acute urinary retention, gross hematuria, lower abdominal pain	9.8	Benign prostatic tissue	Mutilocular, retrovesicular midline mass with multiple thin septations	11 × 9 × 7	NA	Straw-colored fluid	Partial prostatectomy with enucleation of the mass	NA	NA
Rahman et al.	2016	1	74	Hypogastric pain, constipation, obstructive voiding symptom	20.5	Benign prostatic tissue	Multiloculated cystic pelvic mass, peripheral contrast enhancement	11.6 × 9 × 8	NA	Dense brown liquid	Laparoscopic	48 months	No recurrence
Our case	2018	1	50	Abdominal distension, lower urinary symptom	NA	Spindle cell tumor with focal smooth muscle differentiation	Multiloculated cystic mass	32 × 23 × 10	3000	Blackish-brown fluid	Surgical resection	6 months	No recurrence

*NA* not available

Clinical presentation and radiographic features are helpful for preoperative diagnosis. As shown in Table 1, giant multilocular prostatic cystadenoma may occur at any age, with reported patient ages ranging from 23 to 80 years at diagnosis. Presenting symptoms are similar to those of benign prostatic hyperplasia and include incomplete voiding and urinary retention. Almost all cases in the literature noted lower urinary symptoms. Enlargement of the mass also causes abdominal distension and gastrointestinal symptoms. Our case initially presented with complaints of abdominal distension, but a detailed interview revealed these to be lower urinary symptoms. Urinalysis often reveals hematuria and elevated serum PSA levels.

Imaging findings of retroperitoneal tumors are often overlapping, and the first step to understanding the tumors is to divide them into solid or cystic tumors. In addition, their content, precise localization, extent of local invasion, and vascularity may help to define a specific differential diagnosis [22]. CT and MRI scans of the present giant multilocular prostatic cystadenoma revealed a large retroperitoneal solid mass with multilocular cavities that were compressing adjacent organs, especially the bladder and rectum. As it was located between the bladder and rectum, it was presumed to have arisen from the prostate gland. The mass comprised cysts of various sizes and soft tissue components. MRI may provide additional information, and T2-weighted imaging of the cysts can be suggestive of either fat or blood in the content. MRI may also show attachment to the prostate.

Differential diagnoses of a multicystic retroperitoneal tumor can include liposarcoma, leiomyoma with cystic degeneration, lymphangioma, multilocular peritoneal inclusion cyst, phyllodes variant of atypical prostatic hyperplasia, prostatic abscess, and teratoma. Cysts of the lower male genitourinary tract are uncommon. There are also differential diagnoses for cystic lesions in the pelvis, including Müllerian cysts, utricle cysts, and seminal vesicle cysts. However, these are typically smaller than the giant multilocular prostatic cystadenoma and not “multilocular” [23].

Although imaging studies may provide useful information about the extent of the lesion and invasion to adjacent organs, a clear and definitive diagnosis is possible only through histological means. Histologically, a giant multilocular prostatic cystadenoma is composed of multiple cysts in hypocellular fibrous stromal tissue and dense fibrous stroma that corresponds to a focal solid area. It is characterized by cuboidal cells lining the prostate glands and cyst, with positive immunohistochemical staining for PSA in epithelial cells [2]. No previous studies have reported a diagnosis of giant multilocular prostatic cystadenoma preoperatively by biopsy, and biopsy showed benign prostatic glands and stroma in some reports. The biopsy sample of the tumor in our case showed a spindle cell tumor with focal smooth muscle differentiation [2, 24]. If we could diagnose multilocular prostatic cystadenoma before surgery, that is, if we could suspect that the tumor originated from prostate, we might be able to preserve a normal prostate without prostatic urethra injury.

Treatment for giant multilocular prostatic cystadenoma is complete surgical excision. However, adherence to surrounding structures often makes complete resection difficult, and clinical suspicion of malignancy may lead to unnecessarily aggressive surgery and no preservation of urogenital or digestive organs. Despite its benign nature, incomplete resection can result in recurrence [2, 12]. To achieve complete resection, we sacrificed the rectum as well as the left hypogastric nerve and left pelvic plexus, but were able to preserve adjacent organs including the prostate, which resulted in no urinary disorders or sexual dysfunction. Complete resection of giant multilocular prostatic cystadenoma should be considered in light of the surgical trauma involved.

One recent report found that a gonadotropin-releasing hormone antagonist is effective for recurrent giant multilocular prostatic cystadenoma [12], and this may represent a new treatment option for patients with more aggressive or recurrent giant multilocular prostatic cystadenoma.

In conclusion, when a retroperitoneal huge lesion with locular cavities fills the pelvis in men, giant multilocular prostatic cystadenoma should be considered as a differential diagnosis.

## Abbreviations

CT: Computed tomography
MRI: Magnetic resonance imaging
PSA: Prostate-specific antigen
